# Infrared bands of neutral gas-phase carbon clusters in a broad spectral range

**DOI:** 10.1039/d3cp05756a

**Published:** 2024-04-08

**Authors:** Piero Ferrari, Alexander K. Lemmens, Britta Redlich

**Affiliations:** a Radboud University, FELIX Laboratory, Institute for Molecules and Materials 6525 ED Nijmegen the Netherlands piero.ferrariramirez@ru.nl; b Chemical Sciences Division, Lawrence Berkeley National Laboratory Berkeley California 94720 USA

## Abstract

The identification of species in the interstellar medium requires precise and molecule-specific spectroscopic information in the laboratory framework, in broad spectral ranges and under conditions relevant to interstellar environments. In this work, we measure the gas-phase infrared spectra of neutral carbon clusters, C_*N*_ (*N* = 6–11), in a molecular beam. The C_*N*_ distribution is formed by photofragmentation of C_60_ molecules, concurrently showing a top-down formation mechanism. A broad spectral range in the infrared between 500–3200 cm^−1^ (20–3.125 μm) is investigated. We observe strong bands between 5 and 6 μm, in conjunction with novel features in the 3 μm region. Density functional theory calculations reveal that these short wavelength modes correspond to combination bands with significant infrared intensity. Moreover, we identify the *N* ≤ 10 clusters as linear, while C_11_ adopts a ring configuration, placing the linear-to-ring transition at *N* = 11 under our molecular beam conditions. The linearity of C_10_ is discussed based on the formation pathway from larger clusters in energetic conditions. Given the vast and very precise infrared information already been released from the James Webb Space Telescope mission, this infrared spectroscopic data set in conjunction with information on formation mechanisms is of major relevance for identifying neutral carbon clusters in astronomical environments.

## Abstract 2

The gas-phase infrared spectra of neutral carbon clusters are measured in a wide spectral range.

## Graphical abstract

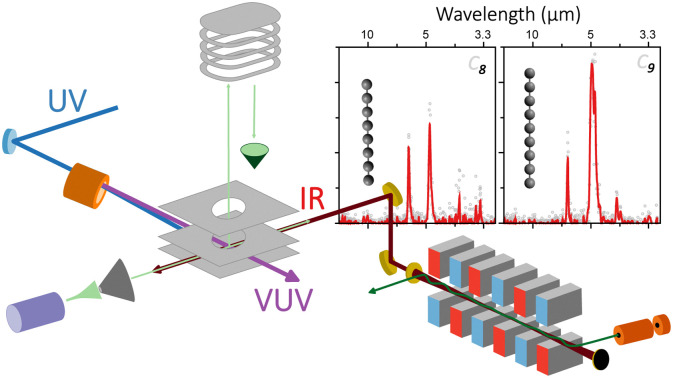

## I. Introduction

The presence of carbonaceous molecules in the interstellar medium (ISM) is undeniable. As of today, more than 240 molecular species have been identified in space, most of which involve one or more carbon atoms.^1^ Still, although many absorption and emission bands have been detected in wide spectral ranges throughout the ISM, only few have been assigned to specific molecular species. Currently, the precise characterization of the chemical composition of the ISM is one of the greatest challenges in astronomy.^2^ With the recent commission of the James Webb Space Telescope (JWST), vast and accurate spectroscopic data from different astronomical environments are being obtained in the mid- and far-infrared spectral ranges, making the collection of relevant spectroscopic information in the laboratory framework a pressing need. In fact, JWST data from, for example, galaxy nuclei,^3^ luminous infrared galaxies,^4^ or star-forming interstellar regions^5^ have already been released.

Particularly relevant are the many unidentified infrared bands seen in different astronomical environments, spanning roughly from 3 to 20 μm.^6^ Over the years, it has been recognized that many of these features are consistent with polycyclic aromatic hydrocarbons (PAHs),^7^ although the exact composition and size of these PAHs remains elusive.^8^ All-carbon species have also been observed in the ISM, including the famous fullerenes C_60_, C_60_^+^ and C_70_,^9,10^ in addition to the small carbon clusters C_2_,^11^ C_3_^12^ and C_5_.^13^ Novel JWST data should provide an even better insight into the possibility that other carbon clusters are present in space. For example, it has been shown that fullerenes can form *via* a top-down approach by the fragmentation of large PAHs in UV irradiated interstellar clouds,^14^ a mechanism that should also lead to the formation of smaller carbon clusters if the environment is sufficiently energetic.^15^

Beyond the relevance in astrochemistry, carbon clusters are fascinating carbon allotropes. For example, their ground-state geometry is known to start as linear moieties, after a critical size in which ring structures are favored, followed by double rings and eventually fullerenes.^16^ Molecular beam studies on C_*n*_^+^^17^ and C_*n*_^−^^18^ species, however, have shown transition sizes that are charge dependent. Moreover, it has been revealed that higher-in-energy isomers can be stabilized under certain conditions, possibly by kinetic trapping. For example, studies have seen C_6_ either in linear or in ring configuration,^19,20^ and linear C_10_ has been observed despite its predicted much higher energy with respect to ring moieties.^21,22^ In this respect, the preferred geometry and spin state of small C_*n*_ clusters remains a subject of interest.^23^ The fragmentation channels of carbon clusters are found to be very rich, with possible fragmentation routes that are size, charge state and isomer specific.^24,25^ Furthermore, several excited carbon clusters have recently been reported to efficiently radiate excess energy *via* recurrent fluorescence,^26^ including C_*n*_^+^ (*n* = 9, 11, 12, 17–27),^27^ C_4_^−^ and C_6_^−^.^28^ The determination of radiation rates involves a detailed understanding of the vibrational energy levels of the clusters,^29^ with anharmonic effects playing a significant, yet majorly unexplored role.^30^

Infrared spectroscopy opens the possibility, on the one hand, to address some of the open questions about the preferred configuration of C_*n*_ clusters, while, on the other hand, provides key information for the search and understanding of the cooling dynamics of pure carbon species in astronomical environments. Over the years, different vibrational modes of C_*n*_ clusters have been investigated, typically in specific spectral ranges per cluster.^31,32^ For example, in Ar matrices the *υ*_4_ and *υ*_5_ stretching modes of linear C_6_,^33^ the *υ*_12_ mode of cyclic C_8_,^34^ and the *υ*_7_ stretching mode of linear C_9_^35^ have been measured. Moreover, in supersonic beams the *υ*_3_ stretching mode of linear C_5_,^36^ the *υ*_4_ and *υ*_5_ stretching vibrations of C_7_,^37–39^ the *υ*_5_ and *υ*_6_ modes of linear C_9_,^40,41^ and antisymmetric stretching of linear C_13_^42^ have been characterized. Nevertheless, systematic information over broad spectral ranges, explored under the same experimental conditions, and for large size distributions are lacking.

In this work, we systematically measured the infrared spectra of a distribution of neutral C_*N*_ (*N* = 6–11) clusters formed by photofragmentation of precursor C_60_ molecules in a molecular beam. The measurements are conducted in the broad infrared range of 500–3200 cm^−1^ (20–3.125 μm), thus covering the far- and mid-infrared regions where JWST operates.

## II. Methods

**Fig. 1:**
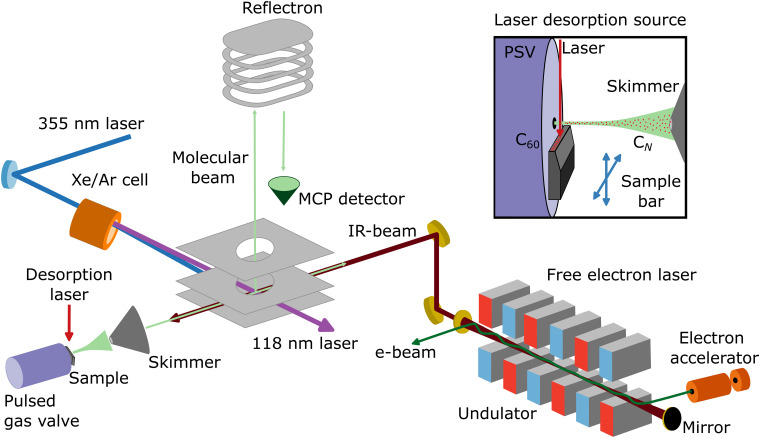
Schematic representation of the molecular beam setup at the FELIX Laboratory (Nijmegen, the Netherlands), including a laser desorption source and a reflectron time-of-flight mass spectrometer. A Xe/Ar cell pumped with the third harmonic of a Nd:YAG laser (355 nm) is used to generate 118 nm laser light, which ionizes a molecular beam of C_*N*_ clusters formed by the desorption and fragmentation of C_60_ molecules, which are detected by an MCP detector. Within the same interaction volume, the counter propagating infrared laser light of the free electron laser FELIX resonantly excites vibrational modes of the clusters. Adapted from ref. 43.

Neutral carbon clusters, C_*N*_ (*N* = 6–11), are formed in a laser desorption source integrated into a molecular beam setup at the FELIX Laboratory in Nijmegen, the Netherlands (see Fig. 1 for a schematic overview of the experimental configuration). To form the C_*N*_ clusters, a powder of C_60_ molecules is deposited on the surface of a movable graphite bar, on which the fundamental of a Nd:YAG laser (1064 nm; 20 mJ pp^−1^) is mildly focused to about 1 mm, desorbing and fragmenting the C_60_ molecules. Before laser desorption, Ar gas at a backing pressure of 3 bar is injected by a pulsed Jordan valve, creating a supersonic molecular beam of neutral C_*N*_ clusters. The molecular beam is collimated by a 2 mm skimmer and enters a reflectron time-of-flight mass spectrometer, with a mass resolution of around *m*/Δ*m* = 2200 at 100 amu. In order to probe the neutral clusters, the third harmonic of a Nd:YAG laser (355 nm) is used to pump a Xe/Ar cell, generating laser light of 118 nm wavelength. Such photons can ionize the C_*N*_ clusters, but only for *N* ≥ 6, because the ionization energy of the smaller clusters is above 10.5 eV.^44^ Therefore, the distribution observed in this work is composed of C_*N*_ clusters with *N* = 6–11. Importantly, specific care was taken to corroborate that the clusters are formed at the source and not by fragmentation at the time-of-flight region by residual 355 nm light. Furthermore, the same experimental procedure was applied using a clean graphite bar that is without C_60_ powder applied, in order to confirm that the carbon clusters are formed by the fragmentation of C_60_ and not by direct ablation from graphite. In this case, no carbon clusters are observed.

To record the infrared spectra, the counterpropagating laser light of the free electron laser FELIX is employed to resonantly excite the vibrational modes of the C_*N*_ clusters, before the neutral species are ionized. By running FELIX at 5 Hz while the molecular beam apparatus is operating at 10 Hz, mass spectra with and without the influence of FELIX are measured alternatingly. The resonant photon absorption is determined as a signal depletion in the cluster mass spectra and therefore, size-dependent information is accessible.

The observation of signal depletion can be the consequence of infrared multiple photon dissociation (IRMPD),^45^ in which each cluster resonantly absorbs multiple infrared photons until the dissociation threshold is overcome, or due to a change in the ionization efficiency of the clusters upon resonant vibrational excitation.^46^ Given that we do not see a clear indication of signal ingrowth upon resonant vibrational excitation, the latter explanation seems more likely. Defining the signal intensity of a cluster with and without FELIX influence by *I* and *I*_0_, respectively, and the FELIX power as *P*, the infrared yield is written as *Y*_IR_ = −ln(*I*/*I*_0_)/*P*. In these experiments, FELIX is scanned over a wide spectral range, 500–3200 cm^−1^ (3.125–20 μm), in steps of 5 cm^−1^. Around the most intense modes a smaller step size of 2 cm^−1^ was employed.

In order to interpret the measured vibrational modes, as well as to determine the geometry of the clusters (linear or ring structures), density functional theory (DFT) calculations were performed using the ORCA 5.03 software package.^47^ For these, the PBE exchange–correlation functional was selected, in combination with the Def2-TZVPP basis set. Linear and ring geometries were optimized, both in singlet and triplet spin states, using the “verytight” convergency criterium for the self consistent field (SCF) cycles and the geometry optimization, as implemented in ORCA. Moreover, a very fine grid size was selected (defgrid3 option). Harmonic vibrational frequencies were computed for all structures, confirming only positive values. Therefore, all geometries correspond to true minima on the potential energy surfaces (PES). Importantly, such frequencies can be directly contrasted with the experimental infrared spectra. Furthermore, overtones and combination bands were calculated, without a direct perturbation of the fundamental frequencies but with intensities determined using the vibrational second-order perturbation theory (VPT2) approach. For the comparison with the experimental data, a common scaling factor of 0.98 was employed.

## III. Results and discussion

**Fig. 2:**
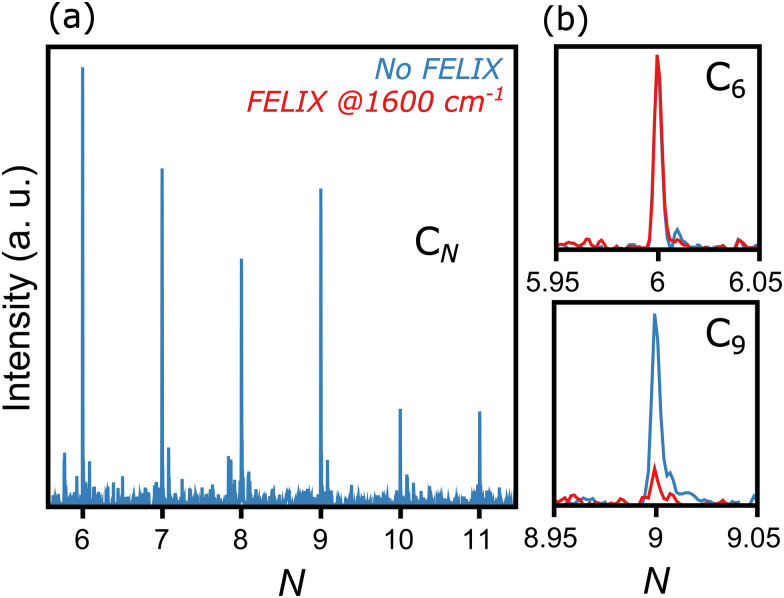
Representative mass spectrum of C_*N*_ clusters ionized by 118 nm laser light, produced by the desorption and fragmentation of C_60_ molecules. Left panel: No FELIX excitation. Right panel: Comparison of intensities without (blue) and with (red) FELIX irradiation at 1600 cm^−1^ for C_6_ (top) and C_9_ (bottom).

Fig. 2 presents a typical mass spectrum of C_*N*_ clusters, in blue without FELIX excitation. In panel (a), the entire size range from *N* = 6 to 11 is depicted. As discussed in Section II, cluster sizes smaller than C_6_ are probably produced in the molecular beam, given the widespread dissociation pathways of carbon clusters,^24,25^ but their high ionization energies prevent their detection in the mass spectrometer. Panels (b) and (c) highlight the influence of resonant photon absorption upon FELIX excitation for the case of 1600 cm^−1^ as an example. Whereas the intensity of C_6_ is not influenced by FELIX, with the curve in red (with FELIX) showing the same intensity as the curve in blue (without FELIX), a clear signal depletion is seen for C_9_, which absorbs infrared photons resonantly. At the maximal FELIX power and the corresponding wavelength, complete depletion is observed for C_9_, showing the good spatial and temporal overlap of FELIX and the molecular beam.

**Fig. 3:**
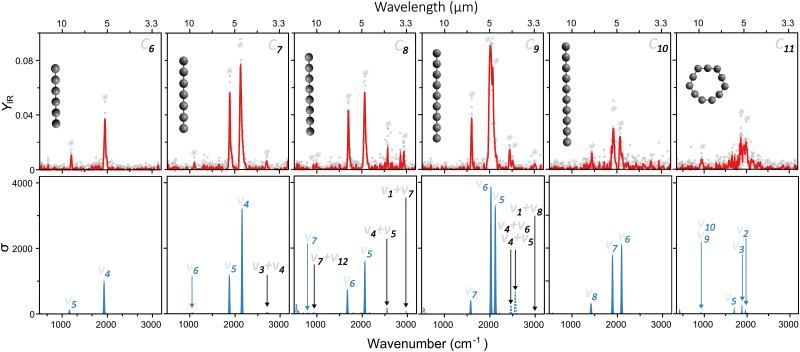
Top: Infrared spectra measured for neutral C_*N*_ clusters in the *N* = 6 to 11 size range. The red curves correspond to a 5-point average of the experimental data points. The detected vibrational bands are marked by asterisks. Bottom: DFT calculated vibrational spectra of the clusters, using the geometries depicted in the top panels. Harmonic frequencies are shown in solid blue, whereas combination bands in dotted lines. Each mode is numbered based on its symmetry, with fundamental transitions in blue and combination bands in black.

Based on the wavelength-dependent depletions, as highlighted in Fig. 2, infrared spectra are constructed. The top panels of Fig. 3 present the measured infrared spectra (*Y*_IR_*versus* FELIX wavelength) of the C_*N*_ (*N* = 6–11) clusters. Several absorption bands are detected for all species, with strong features in the vicinity of 2000 cm^−1^. Given the relevance of these absorption bands for the search of carbon clusters in the ISM, the band centers in μm units are listed in Table 1, based on Gaussian fits of the infrared spectra. Some of the observed features have been characterized in the past, either in noble gas matrices or in molecular beams, but not systematically for wide spectral ranges and for all clusters under the same experimental conditions as provided by this study. Moreover, several bands seen here are new observations, as well as to the best of our knowledge, the spectrum of gas-phase C_11_. In particular, the weaker peaks below 1200 and above 2200 cm^−1^ have not been characterized previously. Interestingly, for C_7_, C_8_ and C_9_, clear features close to 3000 cm^−1^ (3.3 μm) are seen, a spectral region where C–H stretching modes PAHs are detected in astronomical environments.^48^ Here we stress that our clusters do not contain hydrogen, so the observed bands cannot be attributed to C–H stretches.

**Table 1:** Center of the vibrational bands measured for neutral C_*N*_ clusters in the *N* = 6 to 11 size range. Values in μm

C_6_	C_7_	C_8_	C_9_	C_10_	C_11_
8.3	9.1	10.8	6.3	6.9	10.7
5.1	5.3	10.1	5.0	5.2	5.4
	4.7	5.9	4.8	4.8	5.1
	3.7	4.8	4.1		
		3.9	4.0		
		3.4	3.3		

An assignment of the vibrational modes is performed by comparing the measured spectra with those constructed by the DFT calculations. The harmonic frequencies are depicted in the bottom panels in Fig. 3 by solid blue curves, in which a Gaussian function of 20 cm^−1^ full-width-at-half-maximum was used to convolute each vibrational frequency, for visualization purposes. The most intense modes seen experimentally are very well reproduced by the harmonic calculations, using the geometries shown as insets in each panel. The good agreement is not only found for the position of the modes, but also for their relative intensities. This allows us to conclude that from C_6_ to C_10_ the clusters are linear, whereas C_11_ adopts a ring structure.

Regarding the spin configurations, we confidently assign C_7_, C_9_ and C_11_ as singlet states. For C_6_, C_8_ and C_10_ we assign triplet states solely based on energetics, because both spin configurations yield similar infrared spectra, consistent with the experiment. All harmonic frequencies experimentally observed can be ascribed to asymmetric C–C stretches, as labeled in Fig. 3 following the conventional numbering of normal modes.

Interestingly, despite the good computational–experimental agreement, the measured modes below 1200 and above 2200 cm^−1^ are not reproduced by the calculations, irrespective of the employed isomer. Therefore, in addition to the harmonic vibrational frequencies, overtones and combination bands were also calculated. These additional modes are presented in the bottom panels of Fig. 3, marked as dotted lines. Overall, their intensities are weaker than the fundamental modes, as expected, but nevertheless some features are well visible, providing good agreement with the bands yet unexplained by the harmonic calculations. Particularly interesting are the modes seen experimentally at 3.7, 3.4 and 3.3 μm for C_7_, C_8_ and C_9_, respectively, lying on the spectral range where the C–H stretches of PAHs are located.

Based on the analysis in Fig. 3, the linear-to-ring transition in neutral C_*N*_ clusters is found at *N* = 11. While there is consensus that below a critical size C_*N*_ clusters adopt linear geometries, the precise size at which the transition to rings occurs is still under debate, as well as the preferred spin configuration (singlet or triplet).^49,50^ In part, these discussions persist because computations tend to predict different ground state configurations depending on the employed method, including DFT, coupled-cluster or advanced multiconfigurational approaches.^51^

**Fig. 4:**
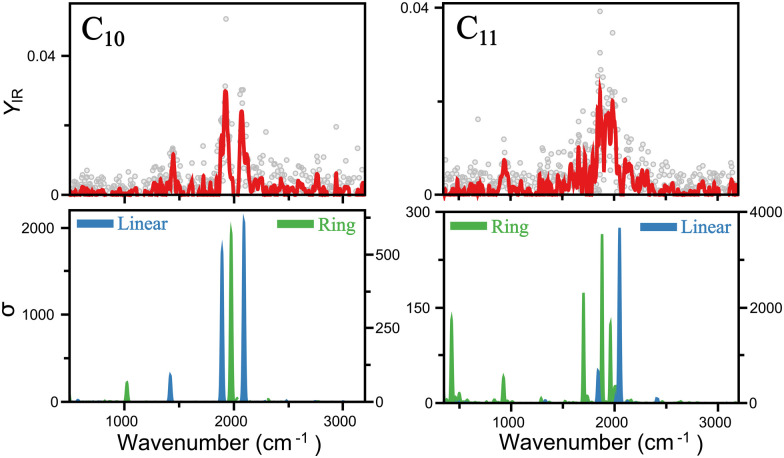
Comparison of the infrared spectra of linear and ring isomers of C_10_ and C_11_. The top panels depict the experimental data. In the bottom panels, the computed spectra of both isomers is shown. Note different left and right *y*-axis, as highlighted by the position of the legends. Linear isomers have higher infrared intensities.

A closer insight into the linear-to-ring transition size is shown in Fig. 4, where the infrared spectra of both isomers–linear and ring - of C_10_ and C_11_ are presented. For C_10_, the two modes clearly distinguished around 2000 cm^−1^ are well reproduced by the linear isomer, in addition to the mode measured at 1447 cm^−1^. In contrast, the ring isomer predicts a band at 1975 cm^−1^, inconsistent with the double mode measured in that range, and in particular, predicts a mode at 1027 cm^−1^ not seen in the experiment. Therefore, we can confidently assign C_10_ as a linear cluster. For C_11_, signal is also seen around 2000 cm^−1^, but individual peaks are less easy to distinguish, suggesting a more congested region of vibrations. This is consistent with the ring isomer, in which three modes are predicted in that region. The linear isomer, in contrast, predicts one very intense mode above 2000 cm^−1^. Moreover, a clear band is detected at 942 cm^−1^, well reproduced by the ring isomer. There is, however, a mode predicted at 422 cm^−1^ that is not detected in the experiment, though at long wavelengths detecting infrared bands can be difficult, in particular if the underlying mechanism is IRMPD, given the low energy per photon. Hence, while in this case is not possible to fully exclude the presence of the linear isomer, there is certainly a contribution for the ring isomer, placing the onset of the linear-to-ring transition at *N* = 11.

**Fig. 5:**
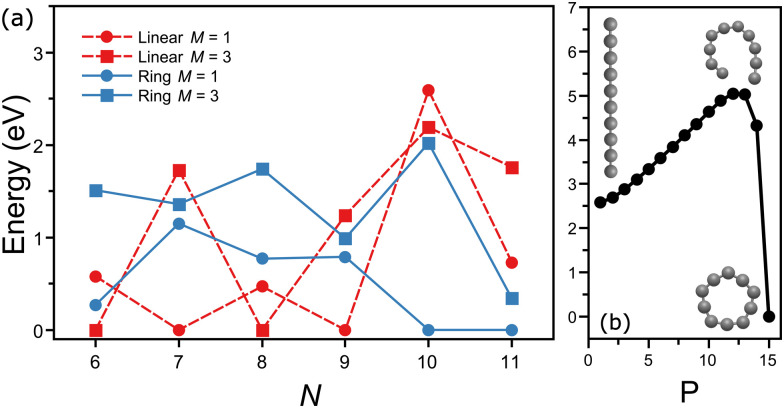
(a) Zpe corrected total energies (pbe/def2-tzvpp) with respect to the lowest-energy configuration of C_*n*_ clusters in linear and ring configurations, in both singlet (*m* = 1) and triplet (*m* = 3) spin states. red (dashed) and blue (continuous) correspond to linear and ring isomers, whereas squares and circles represent singlet and triplet states, respectively. (b) Lowest-energy potential energy surface path connecting the linear and ring configurations of C_10_ in the triplet spin state. *p* represents the points sampled along the trajectory. the *y*-axis is scaled with respect to the energy of the ring isomer.

Interestingly, the energy ordering computed by DFT predicts the ring isomer of C_10_ much lower in energy than the linear configuration. A summary of the total energies calculated at the PBE/Def2-TZVPP level for linear and ring geometries and singlet and triplet spin states for the different C_*N*_ clusters is presented in Fig. 5(a). Overall, DFT finds the following lowest-energy configurations: linear triplet for C_6_, linear singlet for C_7_, linear triplet for C_8_, linear singlet for C_9_, ring singlet for C_10_, and ring singlet for C_11_. In comparison with our assignments based on the matching of the experimental and theoretical IR spectra, all agree except for C_10_, which in our experiment is reliably assigned to the linear isomer, despite that it is nearly 2.5 eV higher in energy.

A simple explanation for the discrepancy between theory and experiment for C_10_ is based on a possible inadequacy of the computational method, although the large energy difference is hard to ignore. Nevertheless, we also performed calculations using the B3LYP functional, yielding a similar energy ordering than with PBE (Fig. 5(a)). Moreover, previous studies employing more advanced wavefunction-based computations also have concluded that the ring isomer of C_10_ is the lowest energy isomer, by about 2 eV.^52^ Another possible explanation is that during the formation process of the clusters the linear isomer of C_10_ is kinetically trapped. While this explanation is more difficult to explore theoretically, it can be of relevance for the understanding of the formation of small carbon clusters in energetic interstellar environments, where formation pathways can involve the destruction of larger fullerenes or even PAHs, following top-down formation schemes. Calculations of the minimum-energy path on the potential energy surface connecting the linear and ring configurations of C_10_ (triplet state) were performed using the nudged elastic band (NEB) method,^53^ implemented in ORCA. As seen in Fig. 5(b), a large energy barrier of almost 2.5 eV is found between both isomers, which could support the idea of kinetic trapping.

In this picture, we start from highly excited C_60_ molecules, which fragment into smaller carbon clusters. The fragmentation pathways are complicated, as they depend on the size, the parent isomer and the internal energy.^25,54^ If C_10_ is formed by the fragmentation of a linear parent, dissociation leads to a linear C_10_, which then needs to overcome a large energy barrier in order to reach the ring configuration. Moreover, if the parent size is a ring cluster, before dissociation occurs the ring must open first, therefore leading to a (quasi)linear parent and also to a linear C_10_ after fragmentation. Importantly, in the experiments presented here this happens during the supersonic expansion, so clusters rapidly cool and can be trapped in local energy minima. The same situation applies to C_11_, but the calculations predict a lower energy barrier connecting the linear and ring configurations (1.5 eV from the linear configuration), so it is more likely that within the time scale of the experiment the lower energy structure is reached. Such a formation pathway could in principle be explored using *ab initio* Born–Oppenheimer molecular dynamics (BOMD) simulations, however, a full exploration of the complex dynamics forming the C_*N*_ clusters from excited C_60_ goes beyond the scope of this work, especially if it also were to involve the supersonic expansion that rapidly freezes the formed species.

Finally, it is worth pointing out that first JWST data has been released, for example, of the star-forming ISM in NGC 7469,^5^ where the IR bands of PAHs are detected. A direct comparison of our IR spectra with the JWST observations, however, is not simple, as the photodissociation regions where bare carbon clusters may be present have not been targeted yet. We therefore present the laboratory spectra aiming at the future identification of these species with upcoming astronomical observations from JWST.

## IV. Conclusions

We presented infrared spectra of neutral carbon clusters, C_*N*_ (*N* = 6–11), formed in a molecular beam by the desorption and photofragmentation of C_60_ molecules. The spectra were recorded in a broad 500–3200 cm^−1^ (3.125–20 μm) spectral range and measured simultaneously for all the species under the same experimental conditions. Our data reveal very intense vibrational modes in the vicinity of 5 μm, in addition to anharmonic modes of significant intensity. Based on the comparison with DFT calculations, we assign all *N* ≤ 10 clusters to linear configurations, whereas C_11_ mainly adopts a ring structure in the molecular beam. Our infrared spectra can be compared with JWST data of different interstellar environments, which may lead to the discovery of these small carbon clusters in space.

## Conflicts of interest

There are no conflicts to declare.

## Supplementary Material
